# On Thompson's Conjecture for Alternating Groups *A*_*p*+3_

**DOI:** 10.1155/2014/752598

**Published:** 2014-07-22

**Authors:** Shitian Liu, Yong Yang

**Affiliations:** School of Science, Sichuan University of Science and Engineering, Zigong, Sichuan 643000, China

## Abstract

Let *G* be a group. Denote by *π*(*G*) the set of prime divisors of |*G*|. Let *GK*(*G*) be the graph with vertex set *π*(*G*) such that two primes *p* and *q* in *π*(*G*) are joined by an edge if *G* has an element of order *p* · *q*. We set *s*(*G*) to denote the number of connected components of the prime graph *GK*(*G*). Denote by *N*(*G*) the set of nonidentity orders of conjugacy classes of elements in *G*. Alavi and Daneshkhah proved that the groups, *A*_*n*_ where *n* = *p*, *p* + 1, *p* + 2 with *s*(*G*) ≥ 2, are characterized by *N*(*G*). As a development of these topics, we will prove that if *G* is a finite group with trivial center and *N*(*G*) = *N*(*A*_*p*+3_) with *p* + 2 composite, then *G* is isomorphic to *A*_*p*+3_.

## 1. Introduction

In this paper, all groups considered are finite and simple groups are nonabelian simple groups. Let *G* be a finite group and let *Z*(*G*) be its center. For any 1 ≠ *x* ∈ *G*, suppose that *x* ^*G*^ denotes the conjugacy classes in *G* containing *x* and *C*_*G*_(*x*) denotes the centralizer of *x* in *G*. We will use *N*(*G*) to denote the set {*n* : *G* has a conjugacy class of size *n*}. Thompson in 1987 gave the following conjecture with respect to *N*(*G*).

*Thompson's Conjecture* (see [1], Question 12.38). If *L* is a finite simple nonabelian group, *G* is a finite group with trivial center, and *N*(*G*) = *N*(*L*), then *G*≅*L*.

Let *π*(*G*) denote the set of all prime divisors of |*G*|. Let *GK*(*G*) be the graph with vertex set *π*(*G*) such that two primes *p* and *q* in *π*(*G*) are joined by an edge if *G* has an element of order *p* · *q*. We set *s*(*G*) to denote the number of connected components of the prime graph *GK*(*G*). A classification of all finite simple groups with disconnected prime graph was obtained in [2, 3]. Based on these results, Thompson's conjecture was proved valid for many finite simple groups with *s*(*G*) ≥ 2 (see [4, 5]). In Ahanjideh and Xu's works, the groups *L*_*n*_(*q*), *D*_*n*_(*q*), *B*_*n*_, *C*_*n*_,  ^2^ *D*_*N*_(*q*), and *E*_7_(*q*) are true for Thompson's conjecture (see [6–10]). Alavi and Daneshkhah proved that the groups *A*_*n*_ with *n* = *p*, *p* + 1, *p* + 2 and *s*(*G*) ≥ 2 are characterized by *N*(*G*) (see [11]). So is there a group with connected prime graph for which Thompson's conjecture would be true? Recently, the groups *A*_10_, *A*_16_, *A*_22_, and *A*_26_ were proved valid for this conjecture (see [12–15]). As a development of these topics, we will prove that Thompson's conjecture is true for the alternating groups *A*_*p*+3_ of degree *p* + 3 with *p* + 2 composite.

We will introduce some notations used in the proof of the main theorem. Let *G* be a group and *p* a prime. Then denote by *G*_*p*_ the Sylow *p*-subgroup of *G*. Let Aut(*G*) and Out(*G*) denote the automorphism and outer automorphism groups of *G*, respectively. Let *ω*(*G*) denote the set of element orders of *G*. The other notations are standard (see [16], for instance).

## 2. Preliminary Results

In this section we will give some preliminary results.

**Lemma 1 .**

Let *x*, *y* ∈ *G*, (|*x*|, |*y*|) = 1, and *xy* = *yx*. Then

1. *C*_*G*_(*xy*) = *C*_*G*_(*x*)∩*C*_*G*_(*y*);
2. |*x* ^*G*^| divides |(*xy*)^*G*^|;
3. if  |*x* ^*G*^| = |(*xy*)^*G*^|, then *C*_*G*_(*x*) ≤ *C*_*G*_(*y*).

**Proof**

See [12, Lemma 1.2] and [7, Lemma 2.3].

**Lemma 2 .**

If *P* and *H* are finite groups with trivial centers and *N*(*P*) = *N*(*H*), then *π*(*P*) = *π*(*H*).

**Proof**

See [12, Lemma 3].

**Lemma 3 .**

Suppose that *G* is a finite group with trivial center and *p* is a prime from *π*(*G*) such that *p* ^2^ does not divide |*x* ^*G*^|  for all *x* in *G*. Then a Sylow *p*-subgroup of *G* is elementary abelian.

**Proof**

See [12, Lemma 4].

**Lemma 4 .**

Let *K* be a normal subgroup of *G*, and $\bar{G}=G/K$.

1. If   $\bar{x}$ is the image of an element *x* of *G* in $\bar{G}$, then $\left|{\bar{x}}^{\bar{G}}\right|$ divides |*x* ^*G*^|.
2. If (|*x* | , |*K*|) = 1, then $C_{\bar{G}}(\bar{x})=C_{G}(x)K/K$.
3. If *y* ∈ *K*, then |*y* ^*K*^| divides |*y* ^*G*^|.

**Proof**

See [12, Lemma 5].

Let exp⁡(*n*, *r*) denote the nonnegative integer *a* such that *r* ^*a*^∣*n* but *r* ^*a*+1^∤*n*.

**Lemma 5 .**

Let *A*_*p*+3_ be the alternating group of degree *p* + 3, where *p* is a prime. Then the following hold.

1. exp⁡(|*A*_*p*+3_ | , 2) = ∑_*i*=1_^*∞*^[(*p* + 3)/2^*i*^] − 1; in particular, exp⁡(|*A*_*p*+3_ | , 2) ≤ *p* + 2.
2. exp⁡(|*A*_*p*+3_ | , *r*) = ∑_*i*=1_^*∞*^[(*p* + 3)/*r* ^*i*^]  for each *r* ∈ *π*(*A*_*p*+3_)∖{2}. Furthermore, exp⁡(|*A*_*p*+3_ | , *r*) < (*p* − 1)/2, where 3 ≤ *r* ∈ *π*(*A*_*p*+3_). In particular, if *r* > [(*p* + 3)/2], then exp⁡(|*A*_*p*+3_ | , *r*) = 1.

**Proof**

(1) By the definition of Gaussian integer function, we have that

(1) exp⁡⁡(|Ap+3|,2)=∑i=1∞[p+32i]−1=([p+32]+[p+322]+[p+323]+⋯)−1≤(p+32+p+422+p+323+⋯)−1=(p+3)(12+122+123+⋯)−1=p+2.

(2) Similarly, as (1), we have that

(2) exp⁡⁡(|Ap+3|,r) ≤(p+3)(1r+1r2+1r3+⋯) =p+3r−1≤p+32

for an odd prime *r* ∈ *π*(*A*_*p*+3_).

If *r* > [(*p* + 3)/2], exp⁡(|*A*_*p*+3_ | , *r*) = 1.

The proof is completed.

Let *S*_*n*_ be the symmetric group of degree *n*. Assume that the cycle has *c*_1_ 1-cycles, *c*_2_ 2-cycles, and so on, up to *c*_*k*_ *k*-cycles, where 1*c*_1_ + 2*c*_2_ + ⋯+*kc*_*k*_ = *n*. Then the number of conjugacy classes in *S*_*n*_ is

(3) $$\begin{array}{c} z=n!{\left(\overset{k}{\underset{i=1}{\prod }}i^{c_{i}}\overset{k}{\underset{i=1}{\prod }}c_{i}!\right)}^{-1}. \end{array}$$

Let *A*_*n*_ be the alternating group of degree *n*.

**Lemma 6 .**

Let *x* ∈ *A*_*n*_. Then, for the size of the conjugacy class *x* ^*G*^ of *x* in *A*_*n*_, one has the following.

1. If, for all even *i*, *c*_*i*_ = 0 and, for all odd *i*, *i* ∈ {0,1}, then |*x* ^*G*^ | = *z*/2.
2. In all other cases, |*x* ^*G*^ | = *z*.

In particular, |*x* ^*G*^ | ≥*z*/2.

**Proof**

See [17].

**Lemma 7 .**

If *n* ≥ 6 is a natural number, then there are at least *s*(*n*) prime numbers *p*_*i*_ such that (*n* + 1)/2 < *p*_*i*_ < *n*. Here

- *s*(*n*) = 6 for *n* ≥ 48;
- *s*(*n*) = 5 for 42 ≤ *n* ≤ 47;
- *s*(*n*) = 4 for 38 ≤ *n* ≤ 41;
- *s*(*n*) = 3 for 18 ≤ *n* ≤ 37;
- *s*(*n*) = 2 for 14 ≤ *n* ≤ 17;
- *s*(*n*) = 1 for 6 ≤ *n* ≤ 13.

In particular, for every natural number *n* > 6, there exists a prime *p* such that (*n* + 1)/2 < *p* < *n* − 1, and, for every natural number *n* > 3, there exists an odd prime number *p* such that *n* − *p* < *p* < *n*.

**Proof**

See Lemma 1 of [18].

**Lemma 8 .**

Let *a*, *b*, and *n* be positive integers such that (*a*, *b*) = 1. Then there exists a prime *p* with the following properties:

- *p* divides *a* ^*n*^ − *b* ^*n*^,
- *p* does not divide *a* ^*k*^ − *b* ^*k*^ for all *k* < *n*,

with the following exceptions: *a* = 2,  *b* = 1; *n* = 6 and *a* + *b* = 2^*k*^; *n* = 2.

**Proof**

See [19].

**Lemma 9 .**

With the exceptions of the relations (239)^2^ − 2(13)^4^ = −1 and (3)^5^ − 2(11)^2^ = 1 every solution of the equation

(4) $$\begin{array}{c} p^{m}-2q^{n}=\pm 1;\;\;p,q\;\;prime;\;\;m,n>1, \end{array}$$

has exponents *m* = *n* = 2; that is, it comes from a unit *p* − *q* · 2^1/2^ of the quadratic field *Q*(2^1/2^) for which the coefficients *p* and *q* are primes.

**Proof**

See [20, 21].

Let *L* be a nonabelian simple group and let* O* denote the order of the outer automorphism group of *L*.

**Lemma 10 .**

Let *L* be a nonabelian simple group. Then the orders and their outer automorphism of *L* are as listed in Tables 1, 2, and 3.

**Proof**

See [22].

## 3. Main Theorem and Its Proof

In this section, we give the main theorem and its proof.

**Theorem 11 .**

Let *G* be a finite group with trivial center and *N*(*G*) = *N*(*A*_*p*+3_) with *p* + 2 composite. Then *G* is isomorphic to *A*_*p*+3_.

**Proof**

We know that *A*_*p*+3_ are characterized by *N*(*G*) if *p* = 7,13,19 (see [12–14]). Then in the following we only consider when *p* ≥ 23.

We divide the proof into the following lemmas.

**Lemma 12 .**

Let *L* = *A*_*p*+3_. Then the following hold.

(1) If 2 ≠ *r* ≤ [(*p* + 3)/2], then we can write *p* + 3 = *kr* + *m* with 0 ≤ *m* < *r* and conjugacy class sizes of *r*-elements of *L* are
  (5) $$\begin{array}{c} \frac{\left(p+3\right)!}{\left(p+3-ir\right)!\cdot r^{i}\cdot i!} \end{array}$$
 for possible *i* with 1 ≤ *i* ≤ *k* = [(*p* + 3)/*r*].
 In particular, if *r* is an odd prime divisor of |*G*|, then conjugacy class sizes of *r*-element of *L* are
  (6) $$\begin{array}{c} \frac{\left(p+3\right)!}{2\cdot k!\cdot r^{2}}, \end{array}$$
 where *p* + 3 = 2*r* + *k* and 0 ≤ *k* < *r*.
(2) If *r* = 2, then one can write *p* + 3 = 2*k* + *m* with 0 ≤ *m* ≤ 1 and conjugacy class sizes of 2-elements of *L* are
  (7) $$\begin{array}{c} \frac{\left(p+3\right)!}{\left(p+3-2i\right)!\cdot 2^{2i}\cdot \left(2i\right)!} \end{array}$$
 for possible *i* with 1 ≤ *i* ≤ *k* = [(*p* + 3)/2].
(3) If *r* > [(*p* + 3)/2], then one can write *p* + 3 = *r* + *m* with 0 ≤ *m* < *r* and conjugacy class sizes of *r*-elements of *L* are
  (8) $$\begin{array}{c} \frac{\left(p+3\right)!}{\left(p+3-r\right)!\cdot r}. \end{array}$$
 In particular, if *r* = *p*, then the conjugacy class size of *p*-elements of *L* is
  (9) $$\begin{array}{c} \frac{\left(p+3\right)!}{6p}. \end{array}$$
(4) The following numbers from *N*(*G*) are maximality with respect to divisibility.
  (a) One of the following holds:
    (*p* + 3)!/2*r* ^2^ if 2 · *r* = *p* + 3;
    (*p* + 3)!/4*r* ^2^ if 2 · *r* + 2 = *p* + 3;
    (*p* + 3)!/2(*k* − 1)*r* ^2^ if 2 · *r* + *k* = *p* + 3 and *k* = 2*n* with *n* ≥ 2;
  (b) (*p* + 3)!/6*p*.

**Proof**

From (3) and Lemma 6, we get the desired results.

**Lemma 13 .**

Let *G* be a finite group with trivial center and *N*(*G*) = *N*(*L*). Then |*L*|∣|*G*| and *π*(*G*) = *π*(*L*).

**Proof**

Note that |*L* | = ∏_*n*∈*N*(*L*)_ *n*. Since |*x* ^*G*^||*C*_*G*_(*x*)| = |*G*|, every member from *N*(*G*) divides the order of *G* and |*L*|∣|*G*|. So by Lemma 12, we have that *π*(*G*) = *π*(*L*).

**Lemma 14 .**

Suppose that *G* is a finite group with trivial center and *N*(*G*) = *N*(*L*). Then the following hold.

1. There exist different primes *r*_1_, *r*_2_, *p* from *π*(*L*) such that *r*_1_, *r*_2_, *p* > [(*p* + 3)/2]. In particular, the Sylow *r*-subgroup *S* of *G* is a cyclic group of order *r* where *r* ∈ {*r*_1_, *r*_2_, *p*}. There does not exist an element of order *r*_1_ · *r*_2_, *r*_1_ · *p*, or *r*_2_ · *p*.
2. For all *n* ∈ *N*(*G*), if *n* is divisible at most by *r* ^*a*^, then the Sylow *r*-subgroup *S* of *G* is of order *r* ^*a*^.

**Proof**

(1) By Lemma 7, there exist different prime numbers *r*_1_, *r*_2_, *p* from *π*(*G*) such that *r*_1_, *r*_2_, *p* > [(*p* + 3)/2].

From Lemmas 12 and 13, we have that the primes *r*_1_, *r*_2_, *p* are prime divisors of |*G*| and *r*_1_^2^, *r*_2_^2^, *p* ^2^ do not divide |*x* ^*G*^| for all *x* ∈ *G*. Then by Lemma 3, *S* is elementary abelian. Therefore if |*x* | = *r*, then |*x* ^*G*^| is an *r*′-number.

Let |*S* | ≥*p* ^2^. Consider an element *y* of *G* with

(10) |yG|=(p+3)!4·r2 if  2r+2=p+3,|yG|=(p+3)!2·r2 if  2r=p+3,

or

(11) |yG|=(p+3)!2(k−1)r2 if  2r+k=p+3,k=2n with  n≥2

by Lemma 12.

Assume that *p*∤|*y*|. Let *x* be an element of *C*_*G*_(*y*) having order *p*. Then *C*_*G*_(*xy*) = *C*_*G*_(*x*)∩*C*_*G*_(*y*), |*x* ^*G*^|∣|(*xy*)^*G*^|, and |*y* ^*G*^|∣|(*xy*)^*G*^| by Lemma 1. Since *S* is abelian, *S* ≤ *C*_*G*_(*x*). Hence, *p*∤|*x* ^*G*^|. It follows that |*x* ^*G*^| equals (*p* + 3)!/6*p* or (*p* + 1)(*p* + 2)(*p* + 3)/3 by Lemma 12.

If |*x* ^*G*^| equals (*p* + 3)!/6*p*, then (*p* + 3)!/6∣|(*xy*)^*G*^|. On the other hand, |*y* ^*G*^|∣|(*xy*)^*G*^|; then we have that

(12) (p+3)!4·r2 ∣ |(xy)G| if  2r+2=p+3,(p+3)!2·r2 ∣ |(xy)G| if  2r=p+3,

or

(13) (p+3)!2(k−1)r2 ∣ |(xy)G| if  2r+k=p+3,k=2n with  n≥2.

Obviously, there is no number from *N*(*G*) such that |*x* ^*G*^ | ∣ | (*xy*)^*G*^| and |*y* ^*G*^ | ∣ | (*xy*)^*G*^|.

Therefore |*x* ^*G*^| equals (*p* + 1)(*p* + 2)(*p* + 3)/3. In the following, we will consider the following three cases.

*Case 1*. |*y* ^*G*^ | = (*p* + 3)!/2 · *r* ^2^ if 2*r* = *p* + 3.

Obviously,  *r*∣(*p* + 1)(*p* + 2)(*p* + 3)/3. Therefore (*p* + 3)!/*r*∣|(*xy*)^*G*^|, a contradiction, since |*x* ^*G*^|∣|(*xy*)^*G*^|, |*y* ^*G*^|∣|(*xy*)^*G*^|, and the maximality of |*y* ^*G*^| = (*p* + 3)!/2 · *r* ^2^.

*Case 2. *|*y* ^*G*^ | = (*p* + 3)!/4 · *r* ^2^ if 2*r* + 2 = *p* + 3.

Obviously, *r*∣(*p* + 1)(*p* + 2)(*p* + 3)/3. Therefore (*p* + 3)!/*r*∣|(*xy*)^*G*^|. Also we get a contradiction as in Case 1.

*Case 3. *|*y* ^*G*^ | = (*p* + 3)!/2(*k* − 1)*r* ^2^ if 2*r* + *k* = *p* + 3 and *k* = 2*n* with *n* ≥ 2.

In this case, *r*∤(*p* + 1)(*p* + 2)(*p* + 3)/3. It follows that |*y* ^*G*^ | = |(*xy*)^*G*^|. By Lemma 1, *C*_*G*_(*y*) ≤ *C*_*G*_(*x*) and so |*x* ^*G*^ | ∣ | *y* ^*G*^|, a contradiction.

Assume that *p*∣|*y*|. Let |*y* | = *p* · *t*. Since *S* is elementary abelian, the numbers *p* and *t* are coprime. Let

(14) $$\begin{array}{c} u=y^{p},\;\;v=y^{t}. \end{array}$$

Then *y* = *uv* and *C*_*G*_(*uv*) = *C*_*G*_(*u*)∩*C*_*G*_(*v*). Therefore,

(15) |vG| ∣ |yG|=(p+3)!2·r2 if  2r=p+3,|vG| ∣ |yG|=(p+3)!2·r2 if  2r+2=p+3,

or

(16) |vG| ∣ |yG|=(p+3)!2(k−1)·r2 if  2r+k=p+3,k=2n, with  n≥2.

On the other hand, the element *v* of *G* is of order *p*. Since the Sylow *p*-subgroup of *G* is elementary abelian, then *p*∤|*v* ^*G*^|. It follows that

(17) $$\begin{array}{c} \left|v^{G}\right|=\frac{\left(p+3\right)!}{6p}\;\text{or}\;\;\frac{\left(p+1\right)\left(p+2\right)\left(p+3\right)}{3} \end{array}$$

by Lemma 12.

If |*v* ^*G*^ | = (*p* + 3)!/6*p*, then |*v* ^*G*^|∣|*y* ^*G*^|, a contradiction. Hence |*v* ^*G*^ | = (*p* + 1)(*p* + 2)(*p* + 3)/3. We consider the following three cases.

*Case 1. *|*y* ^*G*^ | = (*p* + 3)!/2 · *r* ^2^ if 2*r* = *p* + 3.

Obviously,  *r*∣(*p* + 1)(*p* + 2)(*p* + 3)/3. But *r*∤(*p* + 3)!/2*r* ^2^, a contradiction, since |*x* ^*G*^|∣|(*xy*)^*G*^|, |*y* ^*G*^|∣|(*xy*)^*G*^|, and the maximality of |*y* ^*G*^ | = (*p* + 3)!/2 · *r* ^2^.

*Case 2. *|*y* ^*G*^ | = (*p* + 3)!/4 · *r* ^2^ if 2*r* + 2 = *p* + 3.

Obviously,  *r*∣(*p* + 1)(*p* + 2)(*p* + 3)/3. But *r*∤(*p* + 3)!/4*r* ^2^, a contradiction, since |*x* ^*G*^|∣|(*xy*)^*G*^|, |*y* ^*G*^|∣|(*xy*)^*G*^|, and the maximality of |*y* ^*G*^| = (*p* + 3)!/4 · *r* ^2^.

*Case 3. *|*y* ^*G*^ | = (*p* + 3)!/2(*k* − 1)*r* ^2^ if 2*r* + *k* = *p* + 3 and *k* = 2*n* with *n* ≥ 2.

In this case,  *r*∤(*p* + 1)(*p* + 2)(*p* + 3)/3. It follows that |*y* ^*G*^ | = |(*xy*)^*G*^| since the maximality of |*v* ^*G*^|. By Lemma 1, *C*_*G*_(*y*) ≤ *C*_*G*_(*x*) and so |*x* ^*G*^|∣|*y* ^*G*^|, a contradiction.

Therefore the Sylow *p*-subgroup of *G* is of order *p*.

Similarly we can prove the other two cases.

There does not exist an element of order *r*_1_ · *r*_2_, *r*_1_ · *p*, or *r*_2_ · *p*.

(2) Without loss of generality, we assume that *n* is divisible at most by *r* ^2^.

Assume that |*S* | ≥*r* ^3^. Consider an element *x* of *G* such that

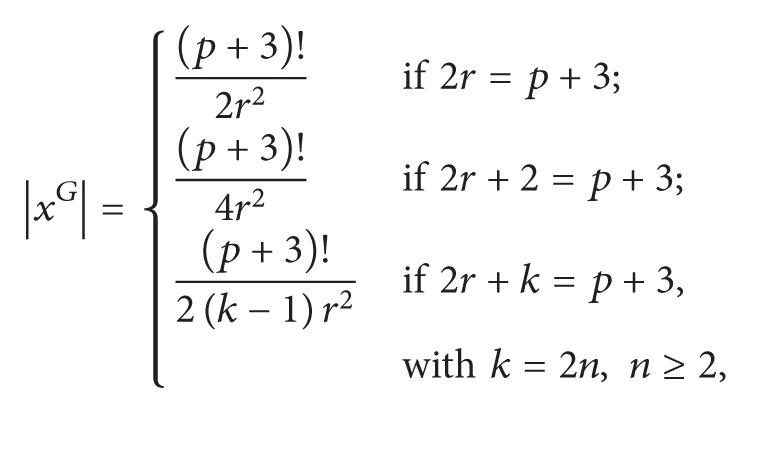
(18)
by Lemma 12.

Let *r*∤|*x*|. Then there is an element *y* of *G* of order *r*. By Lemma 1 we have that *C*_*G*_(*xy*) = *C*_*G*_(*x*)∩*C*_*G*_(*y*), |*x* ^*G*^|∣|(*xy*)^*G*^|, and |*y* ^*G*^|∣|(*xy*)^*G*^|.

If *y* is an *r*-central element, then *S* ≤ *C*_*G*_(*y*) and |*y* ^*G*^| is an *r*′-number. We consider the following three cases.

*Case 1. *2*r* = *p* + 3.

In this case |*y* ^*G*^ | = (*p* + 3)!/2 · *r* ^2^. Then we have that |*x* ^*G*^ | = |*y* ^*G*^ | = |(*xy*)^*G*^| and so *r* ^2^∣∣ | *C*_*G*_(*x*)| = |*C*_*G*_(*y*)| since *y* is an *r*-central element (in fact, if *r* ^3^∣∣ | *C*_*G*_(*x*)|, then there is a conjugacy class size which is a multiple of the number (*p* + 3)!/*r* ^3^ · 3! contradicting 2*r* = *p* + 3). Thus *r*∣|*x* ^*G*^| = |*y* ^*G*^| since *r* ^3^∣|*G*|, a contradiction.

*Case 2. *2*r* + 2 = *p* + 3.

We have that |*y* ^*G*^ | = (*p* + 3)!/4 · *r* ^2^. We similarly can rule out this case as “Case 1: 2*r* = *p* + 3”.

*Case 3. *2*r* + *k* = *p* + 3 and *k* = 2*n* with *n* ≥ 2.

In this case, |*y* ^*G*^ | = (*p* + 3)!/2(*k* − 1)*r* ^2^ or (2*r* + 1)(2*r* + 2) ⋯ (2*r* + *k*)/2(*k* − 2) (we only choose the maximality from the *r*′-number in *N*(*G*)). If the former, then |*x* ^*G*^ | = |*y* ^*G*^ | = |(*xy*)^*G*^| and so *r* ^2^∣∣ | *C*_*G*_(*x*)| = |*C*_*G*_(*y*)| since *y* is an *r*-central element (in fact, if *r* ^3^∣∣ | *C*_*G*_(*x*)|, then there is a conjugacy class size which is a multiple of the number (*p* + 3)!/*r* ^3^ · 3! contradicting 2*r* + *k* = *p* + 3). Thus *r*∣|*x* ^*G*^| = |*y* ^*G*^| since *r* ^3^∣|*G*|, a contradiction. Then |*y* ^*G*^ | = (2*r* + 1)(2*r* + 2)⋯(2*r* + *k*)/2(*k* − 2). But there is no member from *N*(*G*) such that |*x* ^*G*^ | ∣ | (*xy*)^*G*^| and |*y* ^*G*^ | ∣ | (*xy*)^*G*^|.

If *y* is a noncentral *r*-element, then we choose an element *z* of order *p* such that *r* ^2^∣∣ | *z* ^*G*^|. By hypothesis, *r*∣ | *C*_*G*_(*z*)| and, obviously, *r*∤|*z*|. Then by Lemma 1.2 of [4], there is an *r*-element *w* such that 1 ≠ *w* ∈ *C*_*G*_(*z*), *C*_*G*_(*wz*) < *C*_*G*_(*w*), and

(19) $$\begin{array}{c} r\;|\;\frac{\left|C_{G}\left(w\right)\right|}{\left|C_{G}\left(wz\right)\right|}=\frac{\left|{\left(wz\right)}^{G}\right|}{\left|w^{G}\right|}. \end{array}$$

On the other hand, we also have *z* ∈ *C*_*G*_(*w*). Since *wz* = *zw*, then |(*wz*)^*G*^ | = |*z* ^*G*^| and so *C*_*G*_(*z*) ≤ *C*_*G*_(*w*). Therefore *z* ∈ *C*_*G*_(*z*) ≤ *C*_*G*_(*w*). It follows that |*C*_*G*_(*z*)| = |*C*_*G*_(*wz*)| since the maximality of |*z* ^*G*^|, and we get *r*∣|*C*_*G*_(*w*)|/|*C*_*G*_(*wz*)| = 1, a contradiction.

Let *r*∣ | *x*|; then we write |*x* | = *rt*. If *S* is elementary abelian, then (*r*, *t*) = 1. Set

(20) $$\begin{array}{c} u=x^{r},\;\;v=x^{t}. \end{array}$$

Then *x* = *uv* and *C*_*G*_(*x*) = *C*_*G*_(*u*)∩*C*_*G*_(*v*). Hence |*v* ^*G*^ | ∣*x* ^*G*^ and |*u* ^*G*^ | ∣ | *x* ^*G*^|. Since *v* is an element of order *r*, then

(21) |vG|={(p+3)!2r2 if  2r=p+3;(p+3)!4r2 if  2r+2=p+3;(p+3)!2(k−1)r2, or  (2r+1)(2r+2)⋯(2r+k)2(k−2)     if    2r+k=p+3     with    k=2n, n≥2.

If |*v* ^*G*^ | = (*p* + 3)!/2*r* ^2^, (*p* + 3)!/4*r* ^2^, or  (*p* + 3)!/2(*k* − 1)*r* ^2^, then |(*uv*)^*G*^ | = |*v* ^*G*^| and so *r* ^3^∣ | *S* | ∣ | *v* ^*G*^|. It follows that *r*∤|*C*_*G*_(*v*)| = |*C*_*G*_(*uv*)| = |*C*_*G*_(*x*)| contradicts the fact that *x* is an *r*-element. Therefore |*v* ^*G*^ | = (2*r* + 1)(2*r* + 2)⋯(2*r* + *k*)/2(*k* − 2). On the other hand, |*x* ^*G*^ | = (*p* + 3)!/2(*k* − 1)*r* ^2^. Obviously, *r*∣(2*r* + 1)(2*r* + 2)⋯(2*r* + *k*)/2(*k* − 2) but *r*∤(*p* + 3)!/2(*k* − 1)*r* ^2^. It follows that (*p* + 3)!/2(*k* − 1)*r* lies in *N*(*G*), which contradicts the maximality of (*p* + 3)!/2(*k* − 1)*r* ^2^.

Therefore *S* is nonabelian. So we chose an element *z* of order *p* such that *r* ^2^∣ | *z* ^*G*^|. By hypothesis, *r*∣ | *C*_*G*_(*z*)| and, obviously, *r*∤|*z*|. Then by Lemma 1.2 of [4], we also have that |*C*_*G*_(*z*)| = |*C*_*G*_(*wz*)| since the maximality of |*z* ^*G*^|, and we get *r*∣|*C*_*G*_(*w*)|/|*C*_*G*_(*wz*)| = 1, a contradiction.

The lemma is completed.

**Lemma 15 .**

Suppose that *G* is a finite group with trivial center and *N*(*G*) = *N*(*L*). Let *π* = {2,3}. Then *O*_*π*,*π*′_(*G*) = *O*_*π*_(*G*). In particular, *G* is insoluble.

**Proof**

Let *K* = *O*_*π*_(*G*), $\bar{G}=G/K$ and denote by $\bar{x}$ and by $\bar{H}$ the images of an element *x* and a subgroup *H* of *G* in $\bar{G}$, respectively. Assume that the result is not true; then there is a prime *r* ∈ *π*(*L*)∖*π* with $O_{r}(\bar{G})\neq 1$.

Let *r* ∈ {*r*_1_, *r*_2_, *p*} with $O_{r}(\bar{G})\neq 1$. Then $\bar{G}$ contains a Hall {*r* · *s*}-subgroup of order *r* · *s* with *s* ∈ {*r*_1_, *r*_2_, *p*}−{*r*}. However, Hall {*r*, *s*}-subgroup must be cyclic contradicting Lemma 14.

Let *P* be a Sylow *r*-subgroup of $\bar{G}$ where *r* ∈ *π*(*L*)∖{*r*_1_, *r*_2_, *p*}. If $O_{r}(\bar{G})\neq 1$, then $A=Z(O_{r}(\bar{G}))$ is a nontrivial normal subgroup of $\bar{G}$. Let $\bar{x}$ be an element of order *p* in $\bar{G}$. So we have that $\left|{\bar{x}}^{\bar{G}}\right|$ is a divisor of

(22) $$\begin{array}{c} \frac{\left(p+3\right)!}{6p}\;\text{or}\;\frac{\left(p+1\right)\left(p+2\right)\left(p+3\right)}{3}. \end{array}$$

By coprime action lemma, $A=C_{A}(\bar{x})\times [A,\bar{x}]$. In the following, we consider two cases “*r* ≤ [(*p* + 3)/2] and *r* ≥ [(*p* + 3)/2]”.

- *r* > [(*p* + 3)/2] and *r* ≠ *r*_1_, *r*_2_, *p*. In this case, by Lemma 14, we have that the Sylow *r*-subgroup of *G* is of order *r*. Hence there is a Hall {*r*, *p*}-subgroup *H*. Since *H* must be cyclic, then there is an element of order *r* · *p*, a contradiction by the proof of Lemma 14.
- *r* ≤ [(*p* + 3)/2] and *r* ≠ 2,3. If 2*r* = *p* + 3, 2*r* + 2 = *p* + 3, or 2*r* + *k* = *p* + 3 with *k* = 2*n* and *n* ≥ 2, then the index of $C_{A}(\bar{x})$ in *A* is at most *r* ^2^. By Lemma 8, there exists a least divisor *m* of *ϕ*(*p*) such that *p* divides *r* ^*m*^ − 1, and the subgroup $[A,\bar{x}]\langle \bar{x}\rangle$ must be abelian. It follows that $[A,\bar{x}]=1$ and $A=C_{A}(\bar{x})$. If $\bar{y}$ is a nontrivial element of *Z*(*P*)∩*A*, then the order of $C_{\bar{G}}(\bar{y})$ is a multiple of *p*. By Lemma 4, *y* lies in the center of a Sylow *p*-subgroup of *G*. This contradicts Lemma 12. Thus $O_{r}(\bar{G})=1$.

It follows that $O_{r}(\bar{G})=1$ for *r* ∈ {5,7,…, *p*}.

Therefore *O*_*π*,*π*′_(*G*) = *O*_*π*_(*G*). In particular, *G* is insoluble.

**Lemma 16 .**

There is a normal series 1 ≤ *K* ≤ *H* ≤ *G* such that *H*/*K*≅*A*_*p*+3_.

**Proof**

By Lemmas 13 and 14, |*G* | = (*p* + 3)!/2.

By Lemma 15, we have that $H/K\leq \bar{G}\leq \text{Aut}(H/K)$, where *M* : *H*/*K* = *S*_1_ × *S*_2_ × ⋯×*S*_*k*_ is a direct product of nonabelian simple groups *S*_1_, *S*_2_,…, *S*_*k*_. Since *G* cannot contain a Hall {*r*_1_, *r*_2_, *p*}-subgroup, numbers *r*_1_, *r*_2_, and *p* divide the order of exactly one of these groups that is listed as in Lemma 10, and so we assume that they divide *S*_1_. Since $S_{1}⊲\bar{G}$, we let G* and M* denote the factor groups $\bar{G}/S_{1}$ and *M*/*S*_1_, respectively. If *k* > 1, then a Sylow 2-subgroup of G* is nontrivial and its center *Z* has a nontrivial intersection with M*. Consider a nontrivial element *y* of *T* = *S*_2_ × ⋯×*S*_*k*_ such that its image in $\bar{G}$ lies in *Z*. Since *y* centralizes *S*_1_, it lies in the center of a Sylow 2-subgroup of $\bar{G}$ and centralizes an element of order *p*, a contradiction. Thus *M* = *S*_1_, and $\bar{G}$ is almost simple. Therefore

(23) $$\begin{array}{c} \frac{H}{K}\leq \bar{G}\leq \text{Aut}\left(\frac{H}{K}\right). \end{array}$$

Obviously, *r*_1_, *r*_2_, *p*∣ | *H*/*K*| (in fact, if *r*_1_, *r*_2_, *p*∣ | *G*/*H*|, then *r*_1_, *r*_2_, *p*∣ | *G*/*H* | ∣ | Out(*H*/*K*)|, a contradiction, from Lemma 10; if *r*_1_, *r*_2_, *p*∣ | *K*|, then there is an element of order *r* · *p* with *r* ∈ {*r*_1_, *r*_2_} contradicting Lemma 14). In the following, we always assume that *r* ∈ *π*(*G*) = *π*(*L*). In the following, we consider *S*_1_ which is listed as in Tables 1, 2, and 3.

*(i) Case 1.* *H*/*K*≅*A*_*n*_ with *n* ≥ 6.

Then *n* = *p*, *p* + 1,…, *p* + *k* with *p* + 2, *p* + 4,… composite and *p* + *k* + 1 prime. If *k* ≥ 4, then (*p* + *k*)!/2∣(*p* + 3)!, a contradiction. Therefore *H*/*K* is isomorphic to *A*_*p*_,  *A*_*p*+1_, *A*_*p*+2_, or *A*_*p*+3_.

Let *x* be an element of order *p* in *H*. Then |*x* ^*H*^| is *p*′-number since |*H*|_*p*_ = *pH*/*K*≅*A*_*p*_.

Since |*A*_*p*_ | ∣(*p* + 3)!, then 3∣ | *K*|. We have |*x* ^*H*^ | = (*p* − 1)!/2. On the other hand, |*x* ^*G*^ | = (*p* + 3)!/6*p*. It follows that |*x* ^*K*^ | ∣(*p* + 1)(*p* + 2)(*p* + 3)/3 and so there is an element of *r* · *p* or of order *r*′ · *p* with 3 < *r*′ < *r* < *p* and *r* and *r*′ divide one of the prime divisors of the numbers *p* + 1, *p* + 2, or *p* + 3, which contradicts Lemma 14. Similarly, we can rule out these cases “*H*/*K*≅*A*_*p*+1_ and *H*/*K*≅*A*_*p*+2_”.

Therefore *H*/*K*≅*A*_*p*+3_.

*(ii) Case 2.* *H*/*K* is not isomorphic to a sporadic simple group according to Table 3.

*(iii) Case 3.* *H*/*K* is isomorphic to a simple group of Lie type.

Let *q* be a prime power.

(1) *H*/*K*≅*B*_*n*_(*q*) with *n* ≥ 2.
 In this situation, by Lemma 13, *π*(*G*) = {2,3, 5,7,…, *p*} and so
  (24) $$\begin{array}{c} \frac{1}{\left(2,q-1\right)}q^{n^{2}}\overset{n}{\underset{i=1}{\prod }}\left(q^{2i}-1\right)\;|\;\left(p+3\right)!. \end{array}$$
 It follows that *p*∣*q* or *p*∣∏_*i*=1_^*n*^(*q* ^2*i*^ − 1). If *p*∣*q*, then *q* is a power of *p*. Since |*G*_*p*_ | = *p* by Lemma 14, this is impossible as *n* ≥ 2. Therefore *p*∣∏_*i*=1_^*n*^(*q* ^2*i*^ − 1). It follows that *p*∣*q* ^2*t*^ − 1 for some 1 ≤ *t* ≤ *n* as *p* is prime. On the other hand, *q* ^*n*^2^^∣*r* or *q* ^*n*^2^^∣*r* ^*m*^. If the former, then *q* = *r* and *n* = 1, a contradiction. It follows that *q* ^*n*^2^^∣*r* ^*m*^. Hence *r*∣*q* and *m* = *kn* ^2^ for some integer *k* ≥ 1. By Lemma 5, *kn* ^2^ < *p*/2 < (*q* ^2*n*^ − 1)/2, but the equation has no solution in *N*. Furthermore, since *C*_*n*_(*q*) has the same order as *B*_*n*_(*q*), we also can rule out.
(2) *H*/*K*≅*D*_*n*_(*q*) with *n* ≥ 4.
 Therefore we have that (1/(4, *q* ^*n*^ − 1))*q* ^*n*(*n*−1)^(*q* ^*n*^ − 1)∏_*i*=1_^*n*−1^(*q* ^2*i*^ − 1)∣(*p* + 3)!. Since the Sylow *p*-subgroup of *G* is of order *p*, *p*∤*q* as, otherwise, *q* = *p* and thus *n* = 1, a contradiction. It follows that *p*∣*q* ^*n*^ − 1 or *p*∣*q* ^2*t*^ − 1 for some integer 1 ≤ *t* ≤ *n* − 1.
 Let *p*∣*q* ^*n*^ − 1. Then *q* ^*n*(*n*−1)^∣*r* < *p* with *r* prime, and so *q* ^*n*(*n*−1)^ < *p* ^*n*^ − 1. Thus *n* = 1, a contradiction.
 Let *p*∣*q* ^2*t*^ − 1 for some integer 1 ≤ *t* ≤ *n* − 1. Then *q* ^*n*(*n*−1)^∣*r* < *p* with *r* prime, and so *q* ^*n*(*n*−1)^ − 1 ≤ *q* ^2*t*^ − 1 < *q* ^2*n*^ − 1. It follows that *n* = 1,2, 3, a contradiction.
(3) *H*/*K*≅^2^ *A*_*n*_(*q*) with *n* ≥ 2.
 In this situation,
  (25) $$\begin{array}{c} \frac{1}{\left(n+1,q+1\right)}q^{\left(1/2)n(n+1\right)}\overset{n}{\underset{i=1}{\prod }}\left(q^{i+1}-{\left(-1\right)}^{i+1}\right)\;|\;\left(p+3\right)!. \end{array}$$
 Since the Sylow *p*-subgroup of *G* is of order *p* and *n* ≥ 2, we obtain that *p*∣*q* ^(1/2)*n*(*n*+1)^ or *p*∣*q* ^*t*+1^ − (−1)^*t*+1^ for some integer 1 ≤ *t* ≤ *n*.
 If the former, then we have that *q* = *p* and *n* = 1, a contradiction.
 Let *p*∣*q* ^*t*+1^ − (−1)^*t*+1^ for some integer 1 ≤ *t* ≤ *n*. Then *q* ^(1/2)*n*(*n*+1)^∣*r* ^*m*^ for some *m*. If *m* = 1, then *q* = *r* and *n* = 1, a contradiction. It follows that *q* ^(1/2)*n*(*n*+1)^∣*r* ^*m*^ for some *m* > 1 and *r*∣*q*. By Lemma 5, *r* ≤ [*p*/2] < *p*/2 and *n*(*n* + 1)/2 ≤ *m* ≤ *p*/2.
 If *t* is odd, then *p*∣*r* ^*t*+1^ − 1. By Lemmas 8 and 9, *q* = 2, *t* = 5, and *r* = 2. Hence *p* = 3 or 7. If *p* = 3, we can rule out this case since *p* ≥ 5. If *p* = 7, then *n*(*n* + 1) ≤ 3 and so *n* = 1,2, a contradiction, since *t* = 5 < *n*.
 If *t* is even, then *p*∣*r* ^*t*+1^ + 1. So *p*∣*r* + 1 or *p*∣*r* ^*t*^ − *r* ^*t*−1^ + ⋯+1. If the former, then *p*∣*r* + 1 < [*p*/2] + 1, a contradiction. If the latter, then *p* < *r* ^*t*^ + 1. It follows that *p* ≤ *r* ^*t*^ − 1. Let *t* = 2*k* with 1 < *k* < [*p*/2]. Then *p* ≤ *r* ^*k*^ + 1 or *p* ≤ *r* ^*k*^ − 1 and so *p*∣*r* ^*k*^ + 1 or *p*∣*r* ^*k*^ − 1. If *p*∣*r* ^*k*^ − 1, then, by Lemma 8, *r* = 2 and *t* = 5; we also can rule out this case as above. So *p*∣*r* ^*k*^ + 1. It follows that *p*∣*r* ^*t*^ − 1. Similarly, as *p*∣*r* ^*k*^ − 1, we can rule out this case.
(4) *H*/*K*≅*E*_8_(*q*).
 Therefore we have that
  (26) q120(q30−1)(q24−1)(q20−1)(q18−1) ×(q14−1)(q12−1)(q8−1)(q2−1) ∣ (p+3)!.
 It follows that
  (27) p ∣ q120(q30−1)(q24−1)(q20−1)(q18−1)(q14−1) ×(q12−1)(q8−1)(q2−1).
 If *p*∣*q* ^120^, then we can rule out this case since the Sylow *p*-subgroup of *G* is of order *p*. Hence *p*∣*q* ^*t*^ − 1, where *t* ∈ {2,8, 12,14,18,20,24,30}. On the other hand, *r* ^*m*^∣*q* ^120^. If *m* = 1, then *q* = *r* and 1 > 120, a contradiction. If *m* > 1, then *q* = *r* and *m* ≤ 120. By Lemma 5, 120 ≤ *p* and so *p* ∈ {5,7, 11,13,…, 101,103,107,109,113}. It is easy to rule out this case by considering the orders of *G*. Similarly, we can exclude that *H*/*K*≅*E*_6_(*q*), *E*_7_(*q*), and *F*_4_(*q*).
(5) *H*/*K*≅*G*_2_(*q*).
 Then we have *q* ^6^(*q* ^6^ − 1)(*q* ^2^ − 1)∣(*p* + 3)!. It follows that *q* ^6^∣*p*, *p*∣*q* ^6^ − 1, or *p*∣*q* ^2^ − 1.
 If *q* ^6^∣*p*, we rule out this case.
 If *p*∣*p* ^6^ − 1, then there exists a prime *r* such that *q* ^6^∣*r* ^*m*^ for some integer *m*. Therefore *q* = *r* and 6 ≤ *m* < *p* by Lemma 5. It follows that *p* = 5 and so we have a contradiction by considering the order of *G*. Similarly, we also can rule out this case “*p*∣*q* ^2^ − 1”.
(6) *H*/*K*≅^2^ *E*_6_(*q*).
 It is easy to see that (1/(3, *q* + 1))*q* ^36^(*q* ^12^ − 1)(*q* ^9^ + 1)(*q* ^8^ − 1)(*q* ^6^ − 1)(*q* ^5^ + 1)(*q* ^2^ − 1)∣(*p* + 3)!. It follows that *p*∣*q* ^*t*^ − 1 with *t* = 12,8, 6,2, *p*∣*q* ^*k*^ + 1 with *k* = 9,5, or *p*∣*q* ^36^. If *p*∣*q* ^36^, then we rule out this case since the Sylow *p*-subgroup of *G* is of order *p*. So *p*∣*q* ^*t*^ − 1 with *t* = 12,8, 6,2, *p*∣*q* ^*k*^ + 1 with *k* = 9,5, and so there exists a prime *r* such that *q* ^36^∣*r* ^*m*^ for some integer *m*. It means that 36 ≤ *m* ≤ *p*. Therefore *p* = 31,29,23,19,17,13,11,7. We also can rule out this case by order consideration.
(7) *H*/*K*≅^2^ *B*_2_(*q*) with *q* = 2^2*m*+1^.
 It follows that *q* ^2^(*q* ^2^ + 1)(*q* − 1)∣*p*!. Thus *q* ^2^∣*p*, *p*∣*q* ^2^ + 1, or *p*∣*q* − 1.
 If *q* ^2^∣*p*, then we rule out this case.
 If *p*∣*q* ^2^ + 1, then there is a prime *r* such that *q* ^2^∣*r* ^*m*^ and so 2 ≤ *m* ≤ *p* by Lemma 5. Hence *p* = 2, a contradiction. Similarly we can rule out this case “*p*∣*q* + 1”.
 Similarly *H*/*K*≇^2^ *F*_4_(2^2*m*+1^).
(8) *H*/*K*≅^2^ *G*_2_(*q*), *q* = 3^2*n*+1^ with *n* ≥ 1.
 We see that *q* ^3^(*q* ^3^ + 1)(*q* − 1)∣*p*!. Since the Sylow *p*-subgroup of *G* is of order *p*, then *p*∤*q* ^3^. It follows that *p*∣*q* ^3^ + 1 or *p*∣*q* − 1. If *p*∣*q* ^3^ + 1, then there exists a prime *r* such that *q* ^3^∣*r* ^*m*^ for some integer *m*. If *m* = 1, then 1 > 3, a contradiction. Hence 3 ≤ *m* ≤ *p* by Lemma 5, and so *p* = 3, a contradiction. If *p*∣*q* − 1 and *r*∣*q*, then there exists a Frobenius group of *r* · *p* with a kernel of order *r* and a complement of order *p*, respectively, and so there is an element of order *r* · *p*, which contradicts Lemma 14.
(9) *H*/*K*≅^3^ *D*_4_(*q*).
 We have that *q* ^12^(*q* ^8^ + *q* ^4^ + 1)(*q* ^6^ − 1)(*q* ^2^ − 1)∣*p*!. In this case, since *G* has a Sylow *p*-subgroup of order *p*, then *p*∣*q* ^8^ + *q* ^4^ + 1, *q*∣*q* ^6^ − 1, or *p*∣*q* ^2^ − 1. If *p*∣*q* ^8^ + *q* ^4^ + 1, then there exists a prime *r* such that *r* ^*m*^∣*q* ^12^ and so *m* ≤ 12. By Lemma 5, *p* ≤ 12. It follows that *p* = 5,7, 11. Order consideration rules out these cases “*p* = 5,7, 11”. Similarly we can rule out this case “*p*∣*q* ^2^ − 1”.
(10) *H*/*K*≅*A*_*n*_(*q*) with *n* ≥ 1.
 It is easy to get that
  (28) $$\begin{array}{c} \frac{1}{\left(n+1,q-1\right)}q^{n(n+1)/2}\overset{n}{\underset{i=1}{\prod }}\left(q^{i+1}-1\right)\;\;|\;p!. \end{array}$$
 It follows that *p*∣*q* ^*n*(*n*+1)/2^ or *p*∣∏_*i*=1_^*n*^(*q* ^*i*+1^ − 1). If *p*∣*q* ^*n*(*n*+1)/2^, then *n* = 1 since the Sylow *p*-subgroup of *G* is of order *p*, a contradiction. Hence *p*∣∏_*i*=1_^*n*^(*q* ^*i*+1^ − 1) and so *p*∣*q* ^*t*+1^ − 1 for some integer 1 ≤ *t* ≤ *n*. It follows that there exists a prime *r* such that *q* ^*n*(*n*+1)/2^∣*r* ^*m*^ and so *n*(*n* + 1)/2 ≤ *m* ≤ *p*/2 by Lemma 5. Since the Sylow *p*-subgroup of *G* is of order *p*, then *n*(*n* + 1)∣*p* and so *n* = 1, a contradiction.

This completes the proof of the lemma.

**Lemma 17 .**

Consider the following.

*G*≅*A*_*p*+3_.

**Proof**

By Lemma 16,

(29) $$\begin{array}{c} A_{p+3}\leq \bar{G}\leq \text{Aut}\left(A_{p+3}\right)≅S_{p+3}. \end{array}$$

If $\bar{G}≅S_{p+3}$, then there exists an element $\bar{x}$ of $\bar{G}$ with

(30) $$\begin{array}{c} {\bar{x}}^{\bar{G}}=\frac{\left(p+3\right)!}{3p} \end{array}$$

which contradicts Lemma 12.

So $\bar{G}≅A_{p+3}$. Then we define the normal series 1 ≤ *K* ≤ *G* into the chief ones. We prove that *K* = 1. By Lemma 15, *π*(*K*)⊆{2,3}.

If *K* is a 2-group, in this case, let $|\bar{x}|=p$. Then

(31) $$\begin{array}{c} \frac{\left(p+3\right)!}{6p}\;|\;\left|{\bar{x}}^{\bar{G}}\right|. \end{array}$$

By Lemma 12,

(32) $$\begin{array}{c} \left|x^{G}\right|=\left|{\bar{x}}^{\bar{G}}\right|=\frac{\left(p+3\right)!}{6p}. \end{array}$$

So *x* centralizes *K*. It follows that there is an element of 2 · *p* which contradicts Lemma 12 (4).

If *K* is a 3-group. Then similarly as the case “*K* is a 2-group”, we have that $\left|x^{G}|=|{\bar{x}}^{\bar{G}}\right|$ is maximal in *N*(*G*) and *C*_*G*_(*x*) is abelian. So by Lemma 1.12 of [13], *K* ≤ *Z*(*G*) = 1.

Therefore *K* = 1 and *G*≅*A*_*p*+3_.

This completes the proof of the lemma and also of the main theorem.

## 4. Some Applications and Problem

Y. Chen and G. Chen in [23] proved that the group *A*_10_ can be characterized by its order and two special conjugacy classes sizes. Then, obviously, we also have the following result.

**Corollary 18 .**

Let *G* be a finite group with trivial center. Assume that *N*(*G*) = *N*(*A*_*p*+3_) and |*G* | = |*A*_*p*+3_|. Then *G*≅*A*_*p*+3_.

One knows that the alternating groups *A*_*n*_ with *n* = 10,16,22,26 are characterized by *N*(*G*). Then by [4, 5, 15, 21], one has the following.

**Corollary 19 .**

Let *G* be a finite group with trivial center. Assume that *N*(*G*) = *N*(*A*_*n*_) with *n* = *p*, *p* + 1, *p* + 2, *p* + 3. Then *G*≅*A*_*n*_.

## Figures and Tables

**Table 1:** The simple classical groups.

*L*	Lie; rank**L**	*d*	*O*	|*L*|
*L*_*n*_(*q*)	*A*_*n*−1_(*q*)	(*n*, *q* − 1)	2*df*, if *n* ≥ 3;	$\frac{1}{d}q^{n(n-1)/2}\prod_{i=2}^{n}\left(q^{i}-1\right)$
	*n* − 1		*df*, if *n* = 2	
*U*_*n*_(*q*)	^2^ *A*_*n*−1_(*q*)	(*n*, *q* + 1)	2*df*, if *n* ≥ 3	$\frac{1}{d}q^{n(n-1)/2}\prod_{i=2}^{n}\left(q^{i}-{\left(-1\right)}^{i}\right)$
	[*n*/2]		*df*, if *n* = 2	
*PS* *p*_2*m*_(*q*)	*C*_*m*_(*q*)	(2, *q* − 1)	*df*, *m* ≥ 3;	$\frac{1}{d}q^{m^{2}}\prod_{i=1}^{m}\left(q^{2i}-1\right)$
	*m*		2*f*, if *m* = 2	
Ω_2*m*+1_(*q*)	*B*_*m*_(*q*)	2	2*f*	$\frac{1}{2}q^{m^{2}}\prod_{i=1}^{m}\left(q^{2i}-1\right)$
*q* odd	*m*			
*P*Ω_2*m*_^+^(*q*)	*D*_*m*_(*q*)	(4, *q* ^*m*^ − 1)	2*df*, if *m* ≠ 4	$\frac{1}{d}q^{m(m-1)(q^{m}-1)\prod_{i=1}^{m-1}‍(q^{2i}-1)}$
*m* ≥ 3	*m*		6*df*, if *m* = 4	
*P*Ω_2*m*_^−^(*q*)	^2^ *D*_*m*_(*q*)	(4, *q* ^*m*^ + 1)	2*df*	$\frac{1}{d}q^{m(m-1)(q^{m}+1)\prod_{i=1}^{m-1}‍}(q^{2i}-1)$
*m* ≥ 2	*m* − 1			

**Table 2:** The simple exceptional group.

*L*	**L**	*d*	*O*	|*L*|
*G*_2_(*q*)	2	1	*f*, if *p* ≠ 3	*q* ^6^(*q* ^2^ − 1)(*q* ^6^ − 1)
			2*f*, if *p* = 3	
*F*_4_(*q*)	4	1	(2, *p*)*f*	*q* ^24^(*q* ^2^ − 1)(*q* ^6^ − 1)(*q* ^8^ − 1)(*q* ^12^ − 1)
*E*_6_(*q*)	6	(3, *q* − 1)	2*df*	$\frac{1}{d}q^{36}\prod_{i\in \{2,5,6,8,9,12\}}\left(q^{i}-1\right)$
*E*_7_(*q*)	7	(2, *q* − 1)	*df*	$\frac{1}{d}q^{63}\prod_{i\in \{2,6,8,10,12,14,18\}}\left(q^{i}-1\right)$
*E*_8_(*q*)	8	1	*f*	$q^{120}\prod_{i\in \{2,8,12,14,18,20,24,30\}}\left(q^{i}-1\right)$
^2^ *B*_2_(*q*), *q* = 2^2*m*+1^	1	1	*f*	*q* ^2^(*q* ^2^ + 1)(*q* − 1)
^2^ *G*_2_(*q*), *q* = 3^2*m*+1^	1	1	*f*	*q* ^3^(*q* ^3^ + 1)(*q* − 1)
^2^ *F*_4_(*q*), *q* = 2^2*m*+1^	2	1	*f*	*q* ^12^(*q* ^6^ + 1)(*q* ^4^ − 1)(*q* ^3^ + 1)(*q* − 1)
^3^ *D*_4_(*q*)	2	1	3*f*	*q* ^12^(*q* ^8^ + *q* ^4^ + 1)(*q* ^6^ − 1)(*q* ^2^ − 1)
^2^ *E*_6_(*q*)	4	(3, *q* + 1)	2*df*	$\frac{1}{d}q^{36}\prod_{i\in \left\{2,5,6,8,9,12\right\}}\left(q^{i}-{\left(-1\right)}^{i}\right)$

**Table 3:** The simple sporadic groups.

*L*	*d*	*O*	|*L*|
*M*_11_	1	1	2^4^ · 3^2^ · 5 · 11
*M*_12_	2	2	2^6^ · 3^3^ · 5 · 11
*M*_22_	12	2	2^7^ · 3^2^ · 5 · 7 · 11
*M*_23_	1	1	2^7^ · 3^2^ · 5 · 7 · 11 · 23
*M*_24_	1	1	2^10^ · 3^3^ · 5 · 7 · 11 · 23
*J*_1_	1	1	2^3^ · 3 · 5 · 7 · 11 · 19
*J*_2_	2	2	2^7^ · 3^3^ · 5^2^ · 7
*J*_3_	3	2	2^7^ · 3^5^ · 5 · 17 · 19
*J*_4_	1	1	2^21^ · 3^3^ · 5 · 7 · 11^3^ · 23 · 29 · 31 · 37 · 43
*HS*	2	2	2^9^ · 3^2^ · 5^3^ · 7 · 11
*Su* *z*	6	2	2^13^ · 3^7^ · 5^2^ · 7 · 11 · 13
*Mc* *L*	3	2	2^7^ · 3^6^ · 5^3^ · 7 · 11
*Ru*	2	1	2^14^ · 3^3^ · 5^3^ · 7 · 13 · 29
*He*(*F*_7_)	1	2	2^10^ · 3^3^ · 5^2^ · 7^3^ · 17
*Ly*	1	1	2^8^ · 3^7^ · 5^6^ · 7 · 11 · 31 · 37 · 67
*ON*	3	2	2^9^ · 3^4^ · 5 · 7^3^ · 11 · 19 · 31
*Co*_1_	2	1	2^21^ · 3^9^ · 5^4^ · 7^2^ · 11 · 13 · 23
*Co*_2_	1	1	2^18^ · 3^6^ · 5^3^ · 7 · 11 · 23
*Co*_3_	1	1	2^10^ · 3^7^ · 5^3^ · 7 · 11 · 23
*Fi*_22_	6	2	2^17^ · 3^9^ · 5^2^ · 7 · 11 · 13
*Fi*_23_	1	1	2^18^ · 3^13^ · 5^2^ · 7 · 11 · 13 · 17 · 23
*Fi*_24_′	3	2	2^21^ · 3^16^ · 5^2^ · 7^3^ · 11 · 13 · 17 · 23 · 29
*HN*(*F*_5_)	1	2	2^14^ · 3^6^ · 5^6^ · 7 · 11 · 19
*Th*(*F*_3_)	1	1	2^15^ · 3^10^ · 5^3^ · 7^2^ · 13 · 19 · 31
*BM*(*F*_2_)	2	1	2^41^ · 3^13^ · 5^6^ · 7^2^ · 11 · 13 · 17 · 19 · 23 · 31 · 47
*M*(*F*_1_)	1	1	2^46^ · 3^20^ · 5^9^ · 7^6^ · 11^2^ · 13^3^ · 17 · 19 · 23 · 29 · 31 · 41 · 47 · 59 · 71
